# Memantine Attenuates Cocaine and neuroHIV Neurotoxicity in the Medial Prefrontal Cortex

**DOI:** 10.3389/fphar.2022.895006

**Published:** 2022-05-25

**Authors:** Congwu Du, Yueming Hua, Kevin Clare, Kicheon Park, Craig P. Allen, Nora D. Volkow, Xiu-Ti Hu, Yingtian Pan

**Affiliations:** ^1^ Department of Biomedical Engineering, Stony Brook University, New York, NY, United States; ^2^ National Institute on Drug Abuse, Bethesda, MD, United States; ^3^ Department of Microbial Pathogens and Immunity, Rush University Medical Center, Chicago, IL, United States

**Keywords:** NMDA antagonist, addiction, neuroHIV, imaging, prefrontal cortex

## Abstract

Individuals with substance use disorder are at a higher risk of contracting HIV and progress more rapidly to AIDS as drugs of abuse, such as cocaine, potentiate the neurotoxic effects of HIV-associated proteins including, but not limited to, HIV-1 trans-activator of transcription (Tat) and the envelope protein Gp120. Neurotoxicity and neurodegeneration are hallmarks of HIV-1-associated neurocognitive disorders (HANDs), which are hypothesized to occur secondary to excitotoxicity from NMDA-induced neuronal calcium dysregulation, which could be targeted with NMDA antagonist drugs. Multiple studies have examined how Gp120 affects calcium influx and how cocaine potentiates this influx; however, they mostly focused on single cells and did not analyze effects in neuronal and vascular brain networks. Here, we utilize a custom multi-wavelength imaging platform to simultaneously study the neuronal activity (detected using genetically encoded Ca^2+^ indicator, GcaMP6f, expressed in neurons) and hemodynamic changes (measured by total hemoglobin and oxygenated hemoglobin within the tissue) in the prefrontal cortex (PFC) of HIV-1 Tg rats in response to cocaine and evaluate the effects of the selective NMDA antagonist drug memantine on cocaine and HIV neurotoxicity compared to those of non-HIV-1 Tg animals (controls). Our results show that memantine improved cocaine-induced deficit in cerebral blood volume while also attenuating an abnormal increase of the neuronal calcium influx and influx duration in both control rats and HIV-1 Tg rats. Cocaine-induced neuronal and hemodynamic dysregulations were significantly greater in HIV-1 Tg rats than in control rats. With memantine pretreatment, HIV-1 Tg rats showed attenuated cocaine’s effects on neuronal and hemodynamic responses, with responses similar to those observed in control rats. These imaging results document an enhancement of neuronal Ca^2+^ influx, hypoxemia, and ischemia with cocaine in the PFC of HIV-1 Tg rats that were attenuated by memantine pretreatment. Thus, the potential utility of memantine in the treatment of HAND and of cocaine-induced neurotoxicity deserves further investigation.

## Introduction

Drug addiction places individuals at a higher risk for HIV infection and also for rapid progression to neuroHIV (Larrat and Zierler, 1993; Kral et al., 1998; Vignoles et al., 2006). Specifically, cocaine can accelerate the progression of HIV/AIDS as it enhances viral replication and impairs immune responses, targeting macrophages and lymphocytes (Peterson et al., 1990; Peterson et al., 1991). Cocaine might also accelerate the development of HIV-1-associated neurocognitive disorder (HAND), which can manifest as mild cognitive and motor impairment up to dementia and encephalitis (Larrat and Zierler, 1993; Antinori et al., 2007; McArthur et al., 2010). A hallmark of HAND is neuronal degeneration, which is hypothesized to occur due to excitotoxicity from the dysregulation of calcium homeostasis mediated by overactive NMDA receptors and L-type Ca^2+^ channels (Lipton et al., 1991; New et al., 1997; Hu, 2016). The neurotoxic HIV-1 proteins trans-activator of transcription (Tat) and Gp120 are implicated in mediating the dysfunction of NMDA receptors, thus contributing to the abnormally enhanced calcium influx (Nath et al., 1996; Bansal et al., 2000; Nath et al., 2000). Cocaine acts synergistically with HIV-1 Tat and Gp120, potentiating further excessive calcium influx and neurotoxicity (Koutsilieri et al., 1997; Yao et al., 2009). This sudden and prolonged increase in neuronal calcium ([Ca^2+^]_in_) leads to mitochondrial dysfunction, increased reactive oxygen species, and caspase activation (Aksenov et al., 2006; Armada-Moreira et al., 2020). Clinically, patients with HAND have shown attenuated cerebral blood flow including in prefrontal cortex (PFC) regions that might reflect neurotoxicity-related decreases in neuronal activity (Schielke et al., 1990).

Like HAND, preclinical models of cocaine use disorders (CUDs) showed that cocaine alone led to neuronal calcium dysregulation in brain tissues as well as hypoxemia in the PFC (Allen et al., 2019; Du et al., 2021). In combination with the dysregulation of voltage-gated L-type Ca^2+^ channels (Nasif et al., 2005a; Nasif et al., 2005b; Ford et al., 2009; Napier et al., 2014; Wayman et al., 2015; Wayman et al., 2016), NMDA receptors are also pivotal in the development of addictive behaviors and neural adaptations that are mediated by neuronal Ca^2+^ dysregulation. Conditional knockout of these receptors leads to the loss of condition place preference with cocaine administration, while the application of an NMDA antagonist inhibits neuronal spine adaptions in the striatum (Agatsuma et al., 2010; Ren et al., 2010).

Multiple studies have revealed how HIV proteins such as Tat and Gp120 disturb neuronal calcium influx as well as how cocaine potentiates this influx and vice versa (Peterson et al., 1991; Yao et al., 2009; Buch et al., 2011; Wayman et al., 2012; Napier et al., 2014; Wayman et al., 2015; Wayman et al., 2016). Moreover, HIV-1 Tat also facilitates dopamine (DA) neurotransmission in the mesocorticolimbic systems by inhibiting the activity of DA transporters (DATs) (Zhu et al., 2018; Quizon et al., 2021), which is also the target of cocaine’s effects, and could also promote the cocaine-induced dysregulation of intracellular calcium homeostasis. However, many of these studies have been limited to looking at single cells either *in vivo* or *in vitro* and did not analyze the concurrent pathogenesis of hemodynamics, especially in the brain regions that regulate neurocognition and addiction. To study the impact of HIV-1 and its associated proteins on the brain’s reward pathways, we utilized a rodent model of neuroHIV that expresses the HIV-1-associated proteins Tat, Gp120, and several others without a viral load. This rodent model is used in conjunction with a custom-built multi-spectral imaging modality with high spatiotemporal resolution to define the alterations in the prefrontal neuronal and hemodynamic function following exposure to cocaine. Additionally, we also sought to understand how memantine, a selective NMDA antagonist, may modify the PFC’s response to cocaine in both wild-type (non-Tg) and HIV-1 Tg rats. To our knowledge, this is the first neuroimaging study that demonstrates the dysfunction of neuronal activity (i.e., excessive Ca^2+^ influx; [Ca^2+^]_in_) and hemodynamics simultaneously in a brain region regulating neurocognition and addiction in the context of neuroHIV and cocaine use.

## Methods

### Animals and Experimental Design

Experimental protocols utilizing animals were approved by the Stony Brook Institutional Animal Care and Use committee. F344 adult male (wild-type) were acquired from Charles River Lab, and HIV-1 Tg rats were acquired from the University of Maryland, the creator of this neuroHIV rat model (F344 background). The rats were single-housed in a 12-h light/dark cycle and were given *ad libitum* access to food and water. To avoid possible interference of sex hormones on cerebral vasculature and neuronal excitability, we choose to only study male rats, but future studies will evaluate female animals. Animals were divided into experimental groups, as outlined in Table 1.

**TABLE 1:** Animal groups, experimental design, and outcome referring to/comparison.

Experiment	Imaging sets, drug administration	Results referring to	Comparison
**Expt 1**: Monitor NMDA antagonist (memantine)-induced changes in neuronal intracellular calcium and hemodynamics in the PFC of control and HIV-1 Tg rats.	**a.** Control rats (*n* = 3) with memantine administration (10 mg/kg, s.c.); **b.** HIV-1 Tg rats (*n* = 4) with memantine administration (10 mg/kg, s.c.)	Figure 2	
**Expt 2**: Imaged PFC response to cocaine with or without memantine in control rats (*n* = 5).	**a.** First: pretreatment with vehicle followed by cocaine (1 mg/kg, iv); **b.** Second: 2 hours later, pretreatment with memantine (10 mg/kg, s.c.) followed by cocaine (1 mg/kg, iv) 25 min later.	Figures 3–5	Figure 9
**Expt 3**: Imaged PFC response to cocaine with or without memantine in HIV-1 Tg rats (*n* = 5).	**a.** First: pretreatment with vehicle followed by cocaine (1 mg/kg, i.v.); **b.** Second: 2 hours later, pretreatment with memantine (10 mg/kg, s.c.) followed by cocaine (1 mg/kg, i.v.) 25 min later.	Figures 6–8


### Infusion of GCaMP6f Into Rat PFC

**FIGURE 1:**
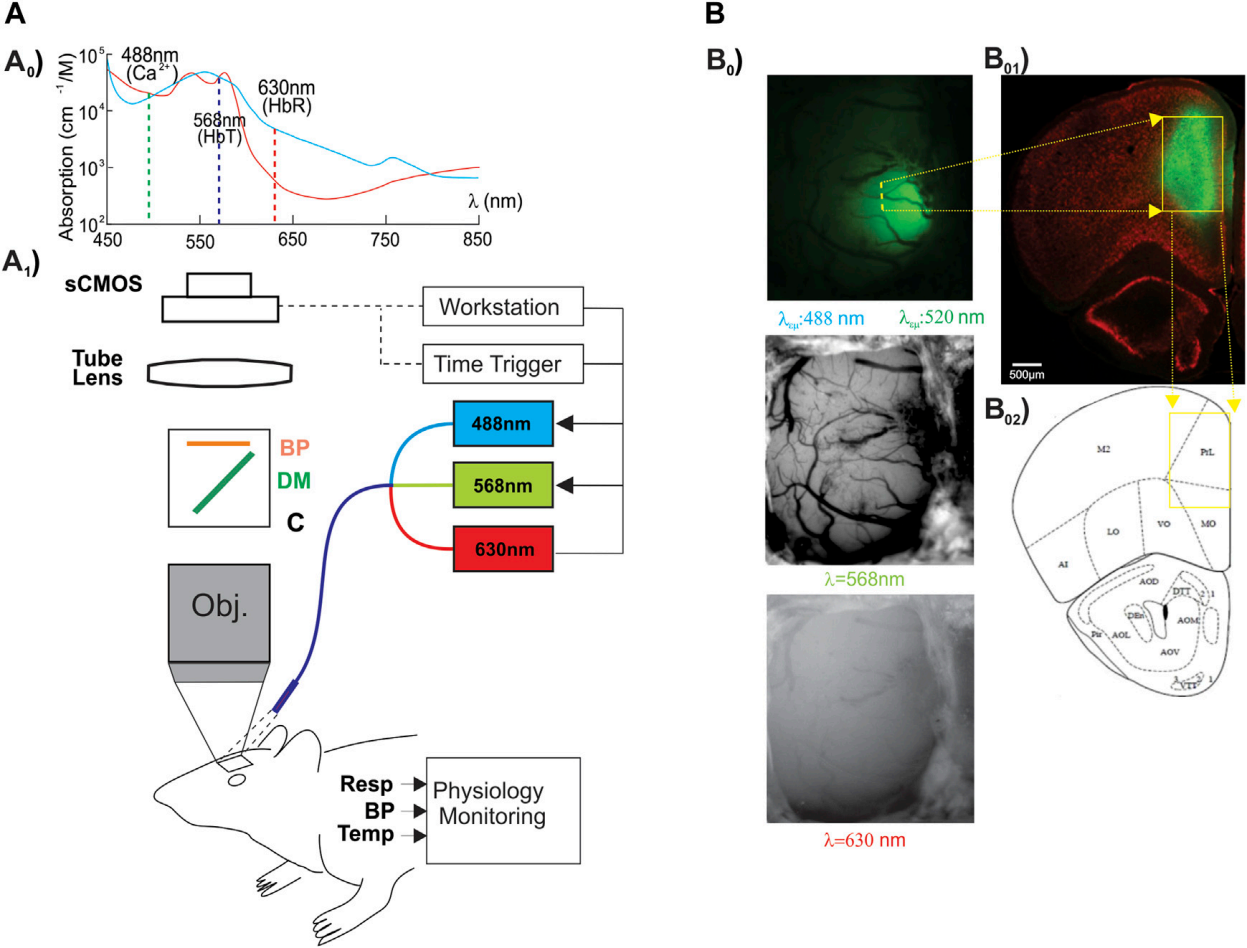
Experimental paradigm and image acquisition. **(A**_**o**_ **)** Hemoglobin (Hb) absorbance spectra. The vertical lines illustrate the center wavelengths of Ca^2+^ fluorescent excitation (λ_ex_ = 488 nm), total hemoglobin (HbT, sensitive at λ_1_ = 568 nm), and deoxygenated-Hb (HbR, sensitive at λ_2_ = 630 nm). **(A**_**1**_ **)** Schematic illustrating the custom multi-wavelength imaging platform (MIP) for the simultaneous capture of neuronal and hemodynamic activities. **(B**_**0**_ **)** Representative in vivo images acquired by the MWIP of the GCaMP6f-expressed Ca^2+^ fluorescence, HbT, and HbR with *ex vivo* confirmation of the GCaMP6f expression in the rat’s PFC (**B**_**01**_). **B**_**02**_ **:** rat brain atlas, showing the location of the GCaMP6f expression in PFC, as shown in a yellow cube (Paxinos and Watson, 1997).

The genetically encoded calcium indicator GCaMP6f was delivered into the PFC of rats to study neuronal activity. Briefly, rats were anesthetized with isoflurane and mounted onto a stereotaxic frame. An incision was made along the scalp, and a hole was drilled into the skull over the PFC area (A/P: +3 mm; M/L:+0.8 mm). A total of 1 μl of AAV5. Syn.gCaMP6f.WPRE.SV40 (Addgene) was infused at two depths (D/V: −1.4mm; D/V: −1.0 mm) at a rate of 0.2 μl/min. Following the infusion of 0.5 μl, a 20-min break occurred to allow for viral diffusion through the tissue. After the surgery, rats were returned to their home cage and observed until awake. Flunixin was given immediately post-operatively and every 12 h thereafter for 3 days. Post-operative recovery was evaluated based on the level of activity as well as food and water intake. Imaging of PFC took place 3–4 weeks after viral delivery. *Ex vivo* imaging was conducted at the end of experiments to confirm the location of GCaMP6f expression (Figure 1B_0_).

### Surgical Preparation for Imaging

Three to four weeks after viral infusion, rats were anesthetized with isoflurane and placed on a ventilator via intubation with a 16-gauge IV catheter into the trachea. Venous and arterial accesses were obtained through the insertion of catheters into the left lower limb femoral artery and vein (0.58 mm ID, 0.99 mm OD, Scientific Commodities Inc.). The arterial line was used to monitor blood pressure, whereas the venous line was used for drug delivery. The rat was then placed in a stereotaxic frame (Kopf 900), and a 4 × 6 mm^2^ craniotomy was performed above the PFC (A/P: 1–5 mm, M/l: −3 to 3 mm). The dura was carefully removed, and 1.25% agarose gel was placed onto the brain surface. A glass coverslip was placed over the agarose gel and secured into place using superglue. During experimentation, the animal’s physiology was recorded including the mean arterial pressure (mean = 82.28, SE = 1.88), body temperature (∼37–38°C), and respiration (∼40–45 breaths per minute) (small Animal Monitoring and Gating System, model 1025L, SA Instruments Inc.).

### Drug Preparation

The NIDA drug supply program provided the cocaine–HCL used in this experiment. Cocaine solutions were freshly made for each experiment by diluting the drug in saline. Memantine (MEM) was purchased from Sigma-Aldrich (St. Louis, MO) and was dissolved in saline (10 mg/ml). For vehicle, saline was utilized.

### Imaging of PFC Through Optical Window

Using a custom-built multi-modality imaging platform (MIP) utilizing previously described methods by our group (Allen et al., 2019; Yuan et al., 2011; Chen et al., 2016), *in vivo* images of the PFC were acquired. As shown in Figure 1, the simultaneous acquisition of [Ca^2+^]_i_, HbR, and HbO_2_ was achieved by the time-sharing approach (Yuan et al., 2011). The light source cycled three LEDs at three different wavelengths (λ_1_ = 568 nm, λ_2_ = 630 nm, and λ_excitation_ = 488 nm; Spectra Light Engine, Lumicor; Figure 1A_0_) at a rate of 1 Hz to illuminate the brain tissue through the cranial window implanted over the PFC (Figure 1A_1_). Images were acquired by a cMOS camera (pixel size: 6.5 μm; Zyla 4.3, Andor) using modified Solis software (version 4.26, Andor). Figure 1B_0_ demonstrates the representative multi-wavelength images obtained from the PFC of a rat *in vivo*. Total hemoglobin (HbT) was calculated based on the changes in HbR and HbO_2_ (Eq. 1).

To determine the effects of memantine on baseline Ca^2+^ and hemodynamics, both HIV-1 Tg and control F344 were given 10 mg/kg s.c. of memantine (Table 1: experiment 1). These animals were first imaged for 5–10 min to acquire a baseline followed by SC injection of memantine (MEM) with an additional 20 min of recording.

Animals in experiments 2 and 3 underwent two sets of imaging with at least 90 min between each cocaine infusion (set **a**: VEH pretreatment followed by cocaine challenge; set **b**: memantine (MEM) pretreatment followed by cocaine challenge). Rats were first (in set **a**) pre-treated with VEH (saline, 1 ml/kg) followed by a 10-min baseline acquisition. Cocaine was infused (1 mg/kg i.v.) over 1 min followed by an additional 60 min of imaging. For the second set of images (in set **b**), rats were pre-treated with 10 mg/kg s.c. memantine. A baseline was acquired over 10 min followed by the administration of acute cocaine challenge over 1 min (1 mg/kg i.v.) with an additional 60-min imaging.

### Processing Acquired Images

To quantify changes in Ca^2+^ and hemodynamics in response to cocaine challenge, we selected five regions of interest (ROI) for each animal (as illustrated in Figure 3: yellow circles). From full-field images, we selected the hemisphere in which GCaMP6f was injected and expressed (Supplementary Figures S2, S3). ROIs were then positioned in regions along the periphery of the GCaMP6f expression and were placed to avoid vasculature, as described in our previous publications (Allen et al., 2019; Du et al., 2020; Du et al., 2021; Park et al., 2022). This ROI positioning criterion was used to minimize the risk of GCaMP6f overexposure following cocaine administration as well as to prevent confounding effects of hemodynamic changes on the measured [Ca^2+^]_i_ fluorescent signal. The same ROIs are used for all three image channels to ensure that the quantification of [Ca^2+^]_i_, HbT, and HbO_2_ was conducted in the same location of the cortex.

Hemodynamic changes (i.e., Δ[HbR] and Δ[HbO_2_]) were calculated using a modified Beer–Lambert law, as outlined in Eq. 1:

$$\left[\begin{array}{c} \Delta HbO_{2}\\ \Delta HbR \end{array}\right]={\left[\begin{array}{cc} \varepsilon_{HbO_{2}}^{\lambda_{1}} & \varepsilon_{HbR}^{\lambda_{1}}\\ \varepsilon_{HbO_{2}}^{\lambda_{2}} & \varepsilon_{HbR}^{\lambda_{2}} \end{array}\right]}^{-1}\left[\begin{array}{c} \ln\left(R_{\lambda_{1}}\left(0\right)/R_{\lambda_{1}}\left(t\right)\right)/L_{\lambda_{1}}\left(t\right)\\ \ln\left(R_{\lambda_{2}}\left(0\right)/R_{\lambda_{2}}\left(t\right)\right)/L_{\lambda_{2}}\left(t\right) \end{array}\right]$$ (1)

Through the MIP, images from illumination with λ_1_ and λ_2_ were captured and the reflectance matrices were determined (R_λ1_(t) and R_λ2_ (t)). Molar excitation coefficients for HbO_2_ and HbR at the given wavelengths are represented by ε^λ1^_HbO2_, ε^λ1^_HbR_, ε^λ2^_HbO2_, and ε^λ2^_HbR_ and L_λ1_ (t) and L_λ2_ (t) are the path lengths of light propagation. When the sum of Δ[HbR] and Δ[HbO_2_] is calculated, the changes in total hemoglobin (Δ[HbT]) can be determined and used as a correlate for total blood volume in the cerebral cortex (Yuan et al., 2011).

Changes in intracellular Ca^2+^ levels were calculated based on the relative change in fluorescent intensity when compared to those in baseline (Δ[Ca^2+^]_i_). Data are represented as the percent change relative to the baseline. To correct for possible absorption in emitted light due to changes in hemodynamics, the fluorescent intensity in the ROIs was normalized to the intensity from areas of non-fluorescence.

### Statistical Analysis

Mean and standard error of the mean were calculated using Microsoft Excel. Statistics were carried out using SigmaStat software (Systat Software INC.). Two-way ANOVAs were used to compare the difference between groups at each time point. Either one-way or two-way ANOVAs with a Bonferroni post hoc test were used for comparison of curve parameters (i.e., peak). *p*<0.05 was considered statistically significant. Raw data and *p*-values are summarized in Supplementary Tables S1–S4.

## Results

### Memantine (NMDA Antagonist) Activates Neuronal [Ca^2+^]_i_, But Has No Effect on Tissue Oxygenation in the PFC of Control and HIV-1 Tg Rats

**FIGURE 2:**
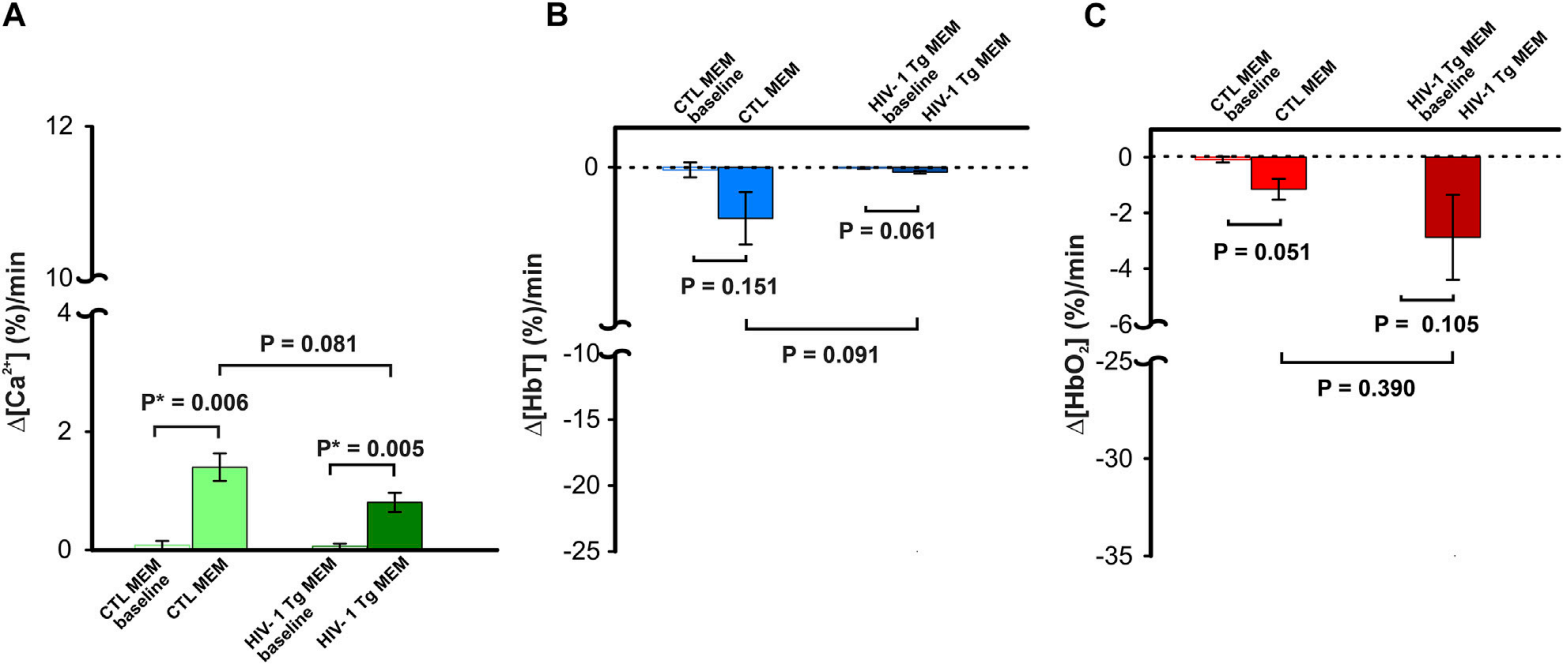
Neuronal Δ[Ca^2+^]_i_ and brain hemodynamic responses to memantine between control and HIV-1 Tg rats. Memantine increased **(A)** neuronal Δ[Ca^2+^]_i_ activity but did not change **(B)** blood volume (Δ[HbT]) nor **(C)** oxygenated hemoglobin (Δ[HbO_2_]) in PFC, and these responses did not differ between controls and HIV-1 Tg rats.

To determine whether memantine would affect neuronal [Ca^2+^]_i_ and hemodynamics (e.g., Δ[HbT], Δ[HbO_2_]) in the PFC, we administered memantine (10 mg/kg, s.c.) into control (F344, *n* = 3) and HIV-1 Tg (*n* = 4) animals. Neuronal Δ[Ca^2+^]_i_ fluorescence, tissue oxygen hemoglobin (Δ[HbO_2_]), and total blood volume (Δ[HbT]) in PFC were detected for 15–20 min (Supplementary Figure S1). Figure 2 summarizes the changes in these three parameters for control and HIV- 1 Tg rats. Figure 2A shows that MEM induced a slight increase in the mean neuronal Δ[Ca^2+^]_i_ to 1.40 ± 0.23 (%/minute, F (1,4) = 29.391, *p* = 0.006) and 0.81 ± 0.16 (%/minute, F (1,6) = 19.126, *p* = 0.005) in PFCs of control and HIV-1 Tg rats, respectively. These changes did not differ between CTL and HIV-1 Tg animals (F (1,5) = 4.756, *p* = 0.081). The mean blood volumes tended to decrease, but these effects were not significant neither in HIV-1 Tg animals (∆[HbT] = -0.062 ± 0.018 (%/min), F (1,6) = 1.28, *p* = 0.061) nor in controls (∆[HbT] = -0.64 ± 0.33 (%/min), F (1,4) = 3.144, *p* = 0.15). Oxygenated hemoglobin did not change in control (∆[HbO_2_] = −1.15 ± 0.37 (%/min), F (1,4) = 7.608 *p* = 0.051) or HIV-1 Tg (∆[HbO_2_] = -2.87 ± 1.52 (%/min), F (1,6) = 3,648, *p* = 0.105) animals. None of the responses to memantine differed significantly between the two groups.

### Memantine Shortened Cocaine-Induced Neuronal [Ca^2+^]_i_ Increases and Reduced Tissue Oxygenation and Total Blood Volumes in Drug-Naïve Animals

Panel B_0_ of Figure 1 shows a representative image of GCaMP6f neuronal expression in the left PFC, where the virus was infused. Fluorescence was primarily expressed in the prelimbic cortex (PrL), which is implicated in cocaine intake (Chen et al., 2013), and reinstatement (Kalivas and McFarland, 2003). To compare cocaine’s effect on PFC with and without NMDA blockade by memantine in the same animal, we administered cocaine twice, first following the vehicle and then after MEM pretreatment (expt 2, Table 1). To minimize the effects of remaining cocaine from the first injection on the response of the second cocaine injection, we waited 2 h between infusions (Du et al., 2021).

**FIGURE 3:**
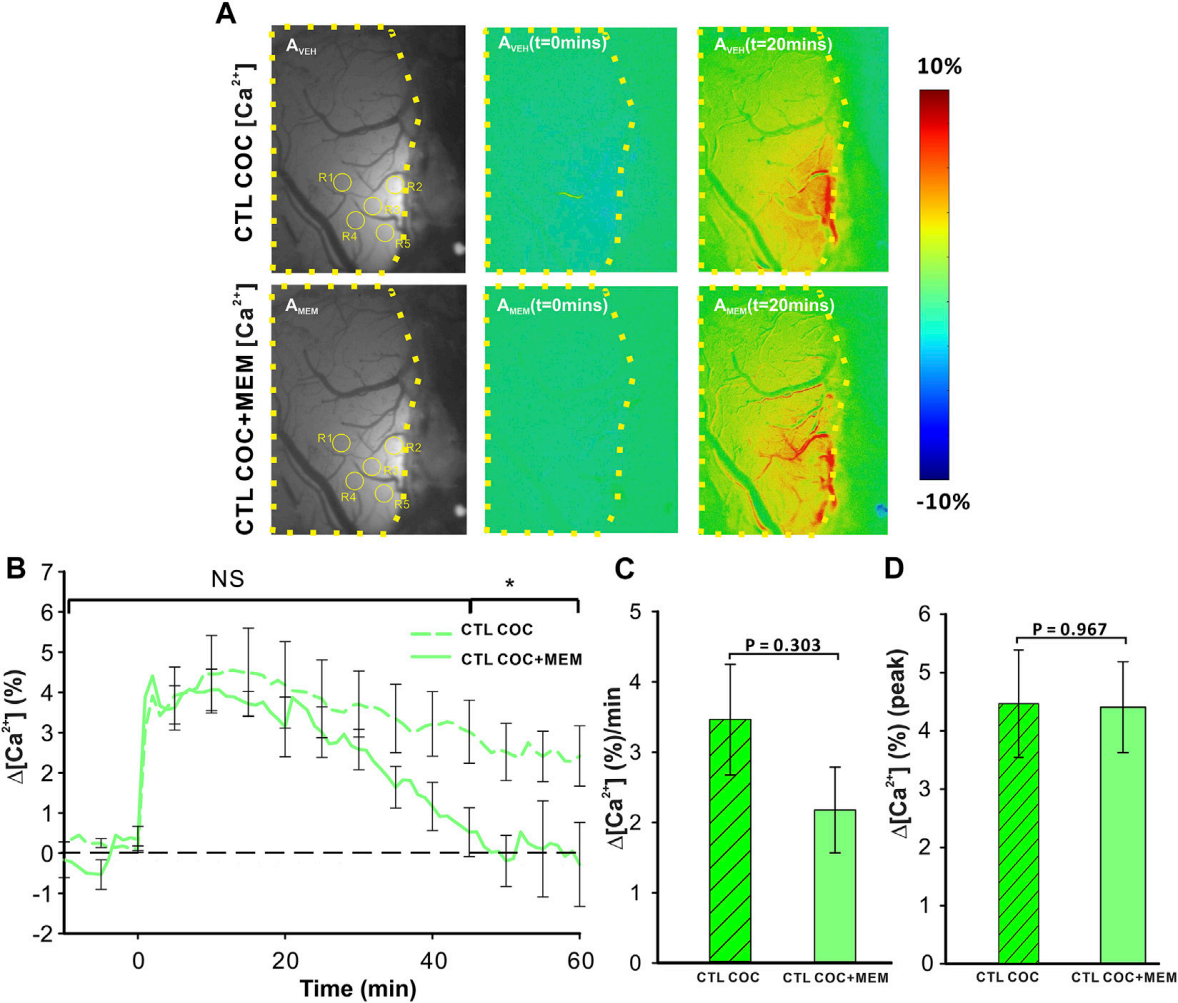
NMDA antagonist (memantine) shortens the duration of [Ca^2+^]_i_ influx in control animals. **(A)** Representative images demonstrating the [Ca^2+^]_i_ fluorescence changes in PFC following cocaine administration (1 mg/kg, i.v.) in vehicle pretreatment (upper panels) and memantine pretreatment (lower panel) rats at t = 20 min relative to the baseline (t = 0 min). Region of brain exposed through the cranial window outlined in yellow, with regions of interest (ROI) for quantification marked with yellow circles. **(B)** Time course of the mean cocaine-induced Δ[Ca^2+^]_i_ response revealing a reduction in the duration of the Ca^2+^ influx following memantine pretreatment. **(C)** Total cocaine-induced Ca^2+^ influx was reduced following memantine pretreatment. **(D)** The presence of memantine did not affect the peak Ca^2+^ influx. Graphed values represent the mean and standard error. NS: not significant *: *p*<0.05, significant difference.

Figure 3A_Veh_ and 3A_MEM_ include representative images of the GCaMP6f neuronal expression before and after MEM infusion, respectively. Figure 3A_veh_ (t = 0 min) and 3A_veh_ (t = 20 min) show the ratio images of GCaMP6f [Ca^2+^]_in_ fluorescence at baseline and 20 min after cocaine without MEM pretreatment, whereas Figure 3A_MEM_(t = 0 min) and 3A_MEM_(t = 20 min) are ratio images of Ca^2+^ fluorescence at baseline and 20 min after cocaine with MEM pretreatment. These images demonstrate that neuronal [Ca^2+^]_i_ fluorescence increases after cocaine with either vehicle or MEM pretreatment. Figure 3B shows the mean time courses of cocaine-induced neuronal [Ca^2+^]_i_ changes in the PFC (*n* = 5) with VEH or MEM-pretreatment, indicating that cocaine triggered an influx in neuronal [Ca^2+^]_i_ in the PFC with different recovery profiles when given after vehicle (cocaine only) or after cocaine with MEM pretreatment (10 mg/kg, s.c.). It illustrates a long-lasting [Ca^2+^]_i_ increase in response to cocaine in VEH animals (i.e., >60 min after cocaine challenge). However, with MEM pretreatment, cocaine-induced Δ[Ca^2+^]_i_ increase recovered at 45–60 min. A two-way ANOVA showed no significant difference between these two-time courses from baseline to 40 min after cocaine (F (13, 84) = 0.706, *p* = 0.753). However, a significant difference was observed from 40 min until the end of recording at 60 min post cocaine (*p* = 0.04). The average rate of Ca^2+^ influx did not differ following memantine pretreatment (F (1, 6) = 1.268, *p* = 0.303; Figure 3C). The peak neuronal ∆[Ca^2+^]_i_ fluorescence increase induced by cocaine corresponded to 4.46 ± 0.92% for vehicle and did not differ from 4.41 ± 0.78% induced with MEM pretreatment (F (1, 6) = 0.00188, *p* = 0.967) (Figure 3D).

**FIGURE 4:**
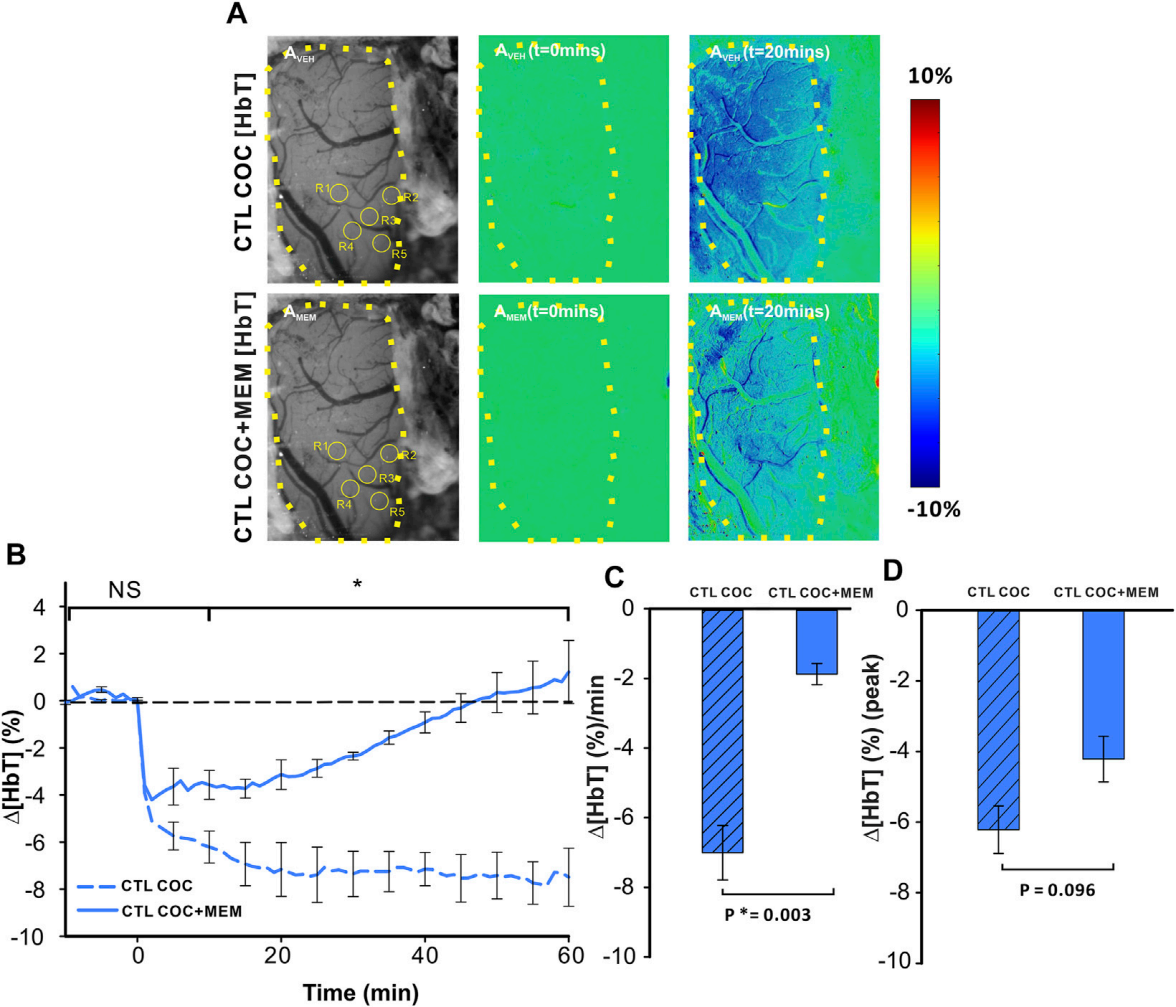
Memantine attenuates cocaine-induced Δ[HbT] decrease in the PFC of control animals. **(A)** Ratio imaging demonstrating the spatiotemporal cocaine-induced reduction in total hemoglobin (Δ[HbT]) in the rat PFC with vehicle (upper panels) and memantine (lower panels) pretreatments. The area encompassed by dashed yellow lines represents the region of brain visualized with the yellow circles indicting the location of ROI for quantification in A_VEH_ and A_MEM._ **(B)** Exposure to memantine prior to cocaine improved cocaine-induced Δ[HbT] reduction as well as shortened the duration of Δ[HbT] response to cocaine. **(C)** Pretreatment with memantine significantly reduced the average Δ[HbT] reduction rate in the PFC. **(D)** Maximal Δ[HbT] decrease (i.e., peak) in response to cocaine did not differ significantly for vehicle and memantine. Graphed values represent the mean and standard error. NS: not significant. *: *p*<0.05, significant difference.

Acute cocaine reduced cerebral blood volume as measured by changes in ∆[HbT] (Figure 4). Figure 4A displays grayscale images with representative images of PFC in a drug-naïve animal at baseline and after MEM pretreatment. Ratio images of PFC before (t = 0 min) and after (t = 20 min) cocaine injection in VEH (upper panels) or MEM pretreatment (lower panel) indicate a decrease in ∆[HbT] in PFC after cocaine (1 mg/kg, i.v.). Comparison between CTL-Coc and MEM-Coc with a two-way ANOVA showed no significant difference within 5 min after cocaine (*p*>0.05) followed by a significant difference from t = 10–60 min post cocaine (F(13, 84) = 5.47, *p*<0.001). Cocaine’s reduction of ∆[HbT] was persistent (i.e., >60 min) after vehicle but returned to baseline after 58.83 ± 14.83 min in the MEM group (Figure 4B). We also compared that cocaine-induced changes in ∆[HbT] integrated over 60 min, which showed significant differences between the vehicle and MEM corresponding to −7.0 ± 0.78% and −1.87 ± 0.31%, respectively (F(1, 6) = 23.075, *p* = 0.003). Figure 4D illustrates the mean peak changes in ∆[HbT] following cocaine, showing no differences between the vehicle and MEM pretreatment (F(1, 6) = 3.887, *p* = 0.096).

**FIGURE 5:**
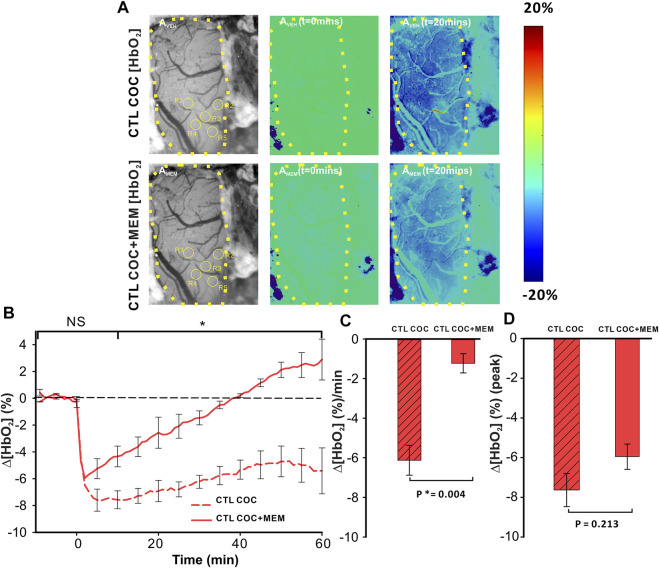
Reduction of Δ[HbO_2_] in the PFC of control animals induced by cocaine is attenuated following memantine pretreatment. **(A)** *In vivo* imaging illustrating the time- and treatment-dependent reduction in oxygenated hemoglobin (HbO_2_) following cocaine exposure in control rats pretreated with vehicle (upper panels) or memantine (lower panel). **(B)** Comparison of the time course for tissue oxygenation showed a faster recovery from cocaine-induced reduction in Δ[HbO_2_] after memantine pretreatment. **(C)** Quantification of the total change in Δ[HbO_2_] indicated that memantine pretreatment significantly improved Δ[HbO_2_] decreases in response to cocaine even though it **(D)** did not significantly change the peak reduction in Δ[HbO_2_]. Graphed values represent the mean and standard error. NS: not significant. *: *p* < 0.05, significant difference.

Cocaine decreased ∆[HbO_2_] in the PFC after vehicle with MEM pretreatment, attenuating the amplitude and duration of the cocaine-induced decrease (Figure 5). Specifically, for VEH, cocaine induced a persistent ∆[HbO_2_] decrease, whereas with MEM pretreatment, the decrease from cocaine returned to baseline at 38 min (t = 1–38 min; *p* < 0.001, t = 38 min; *p* = 0.909) (Figure 5B). Panels A_VEH_ (upper panels in Figure 5A) and A_MEM_ (lower panels in Figure 5A) show representative images of the surface of the PFC with ROI selections and ratio images to illustrate the percent change in [HbO_2_] concentration at t = 0 min and t = 20 min following cocaine infusion (vehicle and MEM pretreatment, respectively). A two-way ANOVA of Δ[HbO_2_] of VEH and MEM showed a significant interaction between pretreatment and time (F(13, 84) = 4.493, *p*<0.001). Point-by-point comparison between these two-time courses showed no differences within 5 min after cocaine between VEH and MEM pretreatment (*p*>0.05) followed by significant differences between them from t = 10–60 min (*p* < 0.05; Figure 5B). Cocaine-induced mean ∆[HbO_2_] change showed a −6.12 ± 0.76 (%/min) reduction with vehicle that was significantly attenuated to −1.23 ± 0.49 (%/min) with MEM (F(1, 6) = 21.023, *p* = 0.004; Figure 5C). The peak cocaine-induced decrease in ∆[HbO_2_] did not differ significantly between vehicle and MEM (−7.63 ± 0.84% with vehicle and −5.95 ± 0.64% with MEM, (F(1, 6) = 1.937, *p* = 0.213; Figure 5D).

### Memantine Depressed Cocaine-Induced Persistent Neuronal [Ca^2+^]_i_ Activity and Reduced Hypoxia in HIV-1 Tg Animals

**FIGURE 6:**
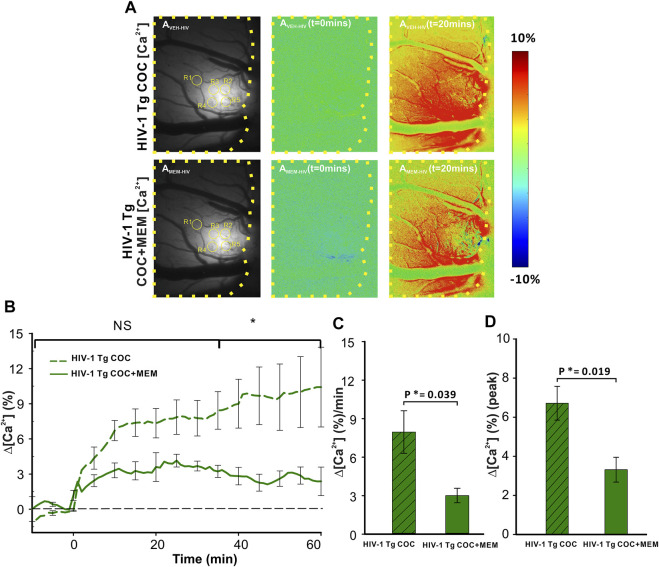
Attenuated neuronal Δ[Ca^2+^]_i_ response to cocaine with memantine pretreatment in HIV-1 Tg rats. **(A)** Representative images of the [Ca^2+^]_i_ fluorescent dynamics in the PFC, illustrating how the response varies spatiotemporally in the PFC of HIV-1 Tg rats. Upper panels represent the response in vehicle-pretreated animals, whereas the lower panel shows the PFC response to cocaine in memantine-pretreated HIV-1 Tg rats. **(B)** Time courses of neuronal Δ[Ca^2+^]_i_ fluorescent changes in both the vehicle and memantine pretreated rats, including the attenuation of cocaine-induced neuronal Δ[Ca^2+^]_i_ with memantine pretreatment. **(C)** Mean Δ[Ca^2+^]_i_ change rate and **(D)** peak of the Δ[Ca^2+^]_i_ signals were significantly reduced in memantine-pretreated rats. Values in graph represent the mean and standard error. The region of the PFC encircled by a yellow line represents the brain region of interest. ROIs quantified are contained within yellow circles in A_VEH-HIV_ and A_MEM-HIV_. NS: not significant. *: *p*<0.05, significant difference.

Cocaine triggered significant increases in neuronal Δ[Ca^2+^]_i_ in PFC of HIV-1 Tg animals that differed when given after vehicle or after MEM pretreatment (Figure 6). Figure 6A_VEH-HIV_ and Figure 6A_MEM-HIV_ show the representative images of GCaMP6f neuronal expression in the PFC of a HIV-1 Tg rat with and without MEM. Figure 6A_VEH_ (t = 0 min) and A_VEH_ (t = 20 min) show the ratio images before and 20 min after cocaine (1 mg/kg, i.v.) for vehicle pretreatment and Figure 6A_MEM-HIV_(t = 0 min) and A_MEM-HIV_(t = 20 min) for MEM pretreatment. A two-way ANOVA showed a significant time effect ([F(13, 84) = 1.701, *p* = 0.072]). Comparing the responses to cocaine at each time point showed no significant difference in Δ[Ca^2+^]_i_ increases from baseline to 30 min (*p*>0.05) between VEH and MEM pretreatment. This is followed by a significant difference after 35 min (p_35-60_<0.05; Figure 6B). The cocaine-induced mean Δ[Ca^2+^]_i_ increase was 7.96 ± 1.66 (%/min) with vehicle, which was significantly reduced to 3.01 ± 0.57 (%/min) with MEM (F(1, 7) = 6.451, *p* = 0.039; Figure 6C). Also, the peaks of ∆[Ca^2+^]_i_ in responses to cocaine differed significantly, corresponding to 6.71 ± 0.86% and 3.32 ± 0.63% for vehicle and MEM, respectively (F(1, 7) = 9.102, *p* = 0.019) (Figure 6D).

**FIGURE 7:**
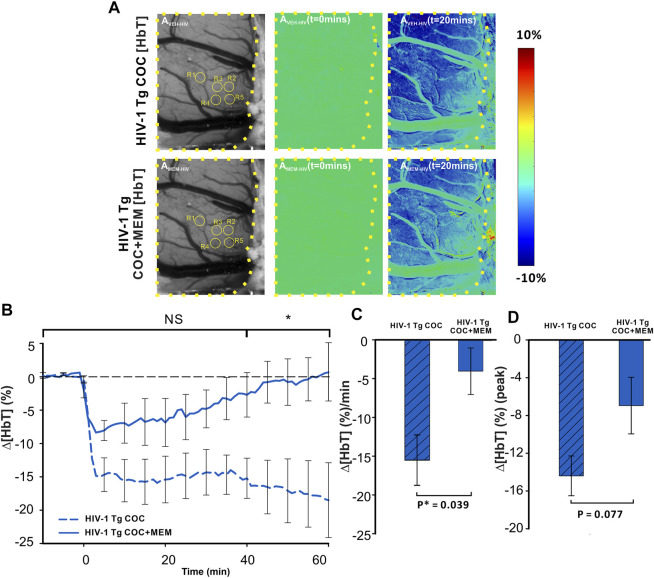
Memantine improves cocaine-induced decrease in Δ[HbT] in HIV-1 Tg rats. **(A)** Spatiotemporal changes of Δ[HbT] in the PFC of HIV-1 Tg rats in response to cocaine with either pretreatment with vehicle (upper panel) or with memantine (lower panel). **(B)** Time course of Δ[HbT] response to cocaine in PFC, illustrating that HIV-1 Tg rats pretreated with memantine returned to baseline within 60 min but not with vehicle pretreatment. **(C)** Memantine attenuated cocaine-induced reductions in Δ[HbT] and **(D)** peak of Δ[HbT] changes. Graphed values represent the mean with standard error. ROIs for quantification are encircled in yellow. The brain region of interest is encompassed by a yellow dashed line. NS: not significant. *: *p*<0.05, significant difference.

Cocaine decreased Δ[HbT] in the PFC of HIV-1 Tg animals with both vehicle and MEM pretreatments (Figure 7A). A two-way ANOVA for the comparison between CTL-Coc and MEM-Coc showed a significant interaction of pretreatment and time ([F(13, 84) = 1.236, *p* = 0.27]). Cocaine’s reduction of ∆[HbT] was persistent with vehicle but recovered at 58.78 ± 26.91 min with MEM pretreatment (Figure 7B). The mean ∆[HbT] was −15.49 ± 3.2 (%/min) and −4.04 ± 3.01 (%/min) for vehicle and MEM pretreatment, respectively (F(1, 7) = 6.418, *p* = 0.039). There was no significant difference in the cocaine-induced peak of ∆[HbT] between vehicle (−14.83 ± 2.41%) and MEM (−6.95 ± 2.3%) (F(1, 7) = 4.306, *p* = 0.077; Figure 7D).

**FIGURE 8:**
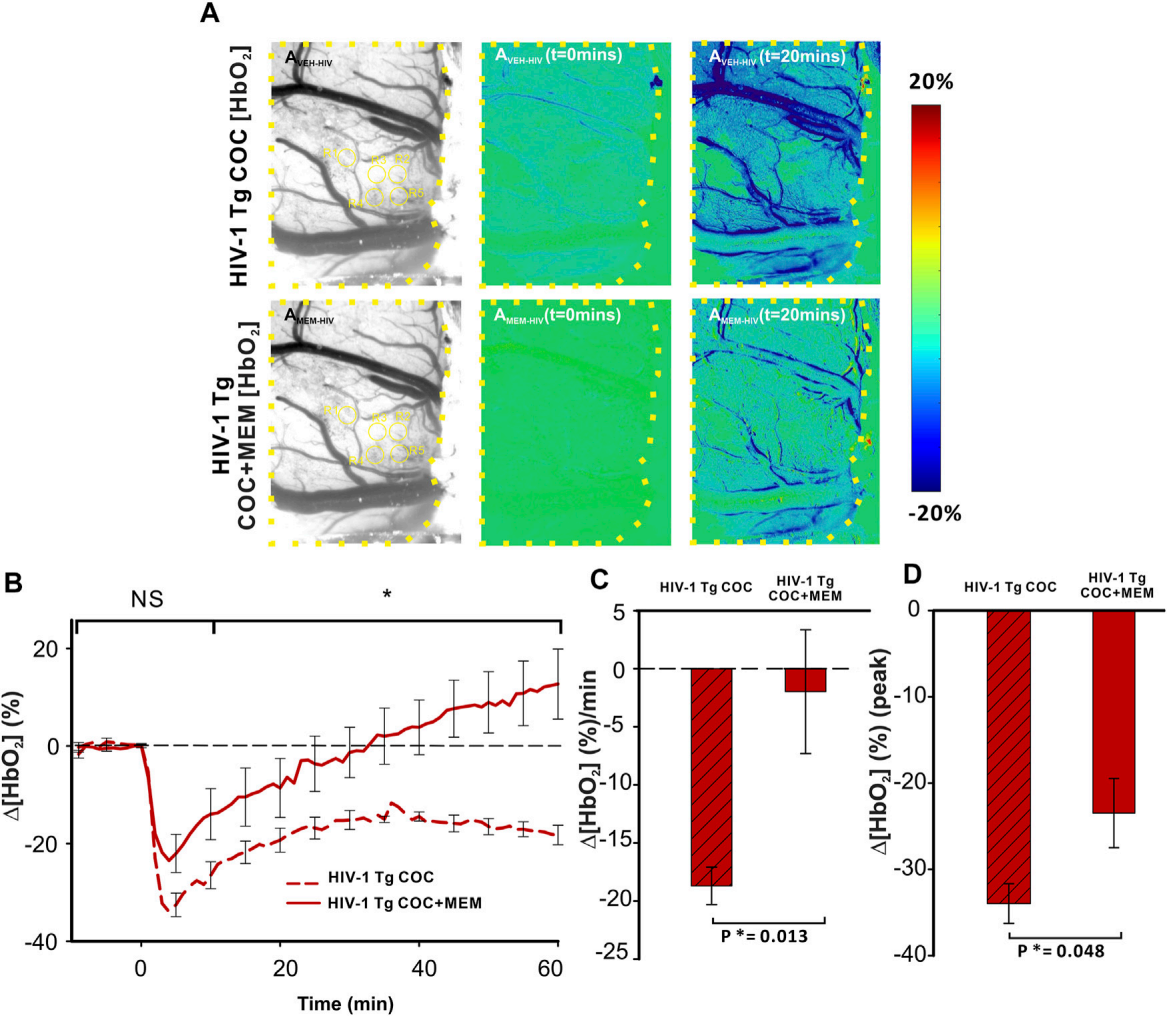
Cocaine-induced decrease in oxygenated hemoglobin improved with memantine in HIV-1 Tg rats. **(A)** Ratio imaging of HbO_2_ in the PFC of HIV-1 Tg rats following exposure to cocaine after pretreatment with either vehicle (upper panel) or memantine (lower panel). Areas encompassed within the dashed yellow lines represent the brain region of interest. ROIs used for quantification are in yellow circles. **(B)** Memantine administration prior to cocaine improved the cocaine-induced reduction in Δ[HbO_2_] returning to baseline but remained decreased in the PFC of animals with vehicle pretreatment. **(C,D)** Total cocaine-induced reduction in Δ[HbO_2_] and peak was significantly improved when HIV-1 Tg rats were pretreated with the NMDA antagonist. NS: not significant. *: *p*<0.05, significant difference.

Cocaine reduced Δ[HbO_2_] in the PFC of HIV-1 Tg animals with vehicle or MEM pretreatment (Figure 8). Similar to memantine’s attenuation of the reduction of cerebral blood volume (∆[HbT]; Figure 7), oxygenated hemoglobin in PFC was also reduced with MEM pretreatment (Figure 8). With vehicle, cocaine decreased Δ[HbO_2_] to −33.97 ± 2.3% at t = 4 min and with memantine to −23.48 ± 4.0% (F(1, 7) = 5.714, *p* = 0.048; Figure 8D). Additionally, cocaine induced a persistent decrease in ∆[HbO_2_] over 60 min with vehicle, whereas with MEM pretreatment, the decrease recovered at 34.93 ± 12 min followed by an overshoot (Figure 8B). The mean changes in cocaine-induced ∆[HbO_2_] were significantly reduced from −18.7 ± 1.6 (%/min) with vehicle to −1.98 ± 5.3 (%/min) with MEM pretreatment (F(1, 7) = 11.066, *p* = 0.013; Figure 8C).

### Cocaine-Induced Neuronal [Ca^2+^]_i_ and Hemodynamic Dysregulations in the PFC Between HIV-1 Tg Rats and Controls, With Memantine Reducing Cocaine’s Effect on Neural and Hemodynamic Changes in PFCs of HIV-Tg Rats

**FIGURE 9:**
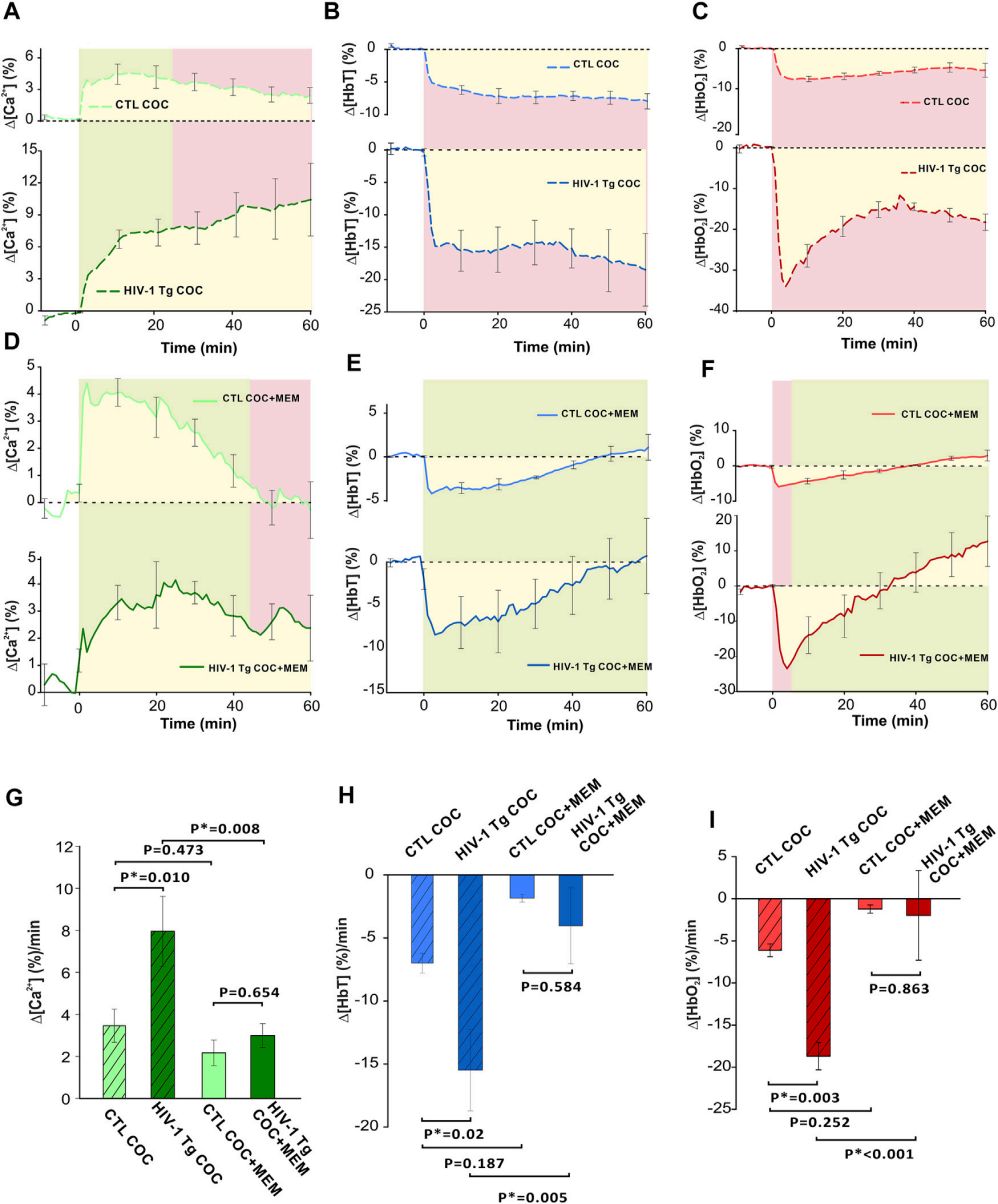
Comparison of cocaine-induced changes between control and transgenic HIV-1 Tg animals with saline or memantine pretreatment. **(A)** Time course of neuronal response to cocaine in HIV-1 Tg and control (F344 non-Tg) rats showing the variation in response to cocaine (1 mg/kg, i.v.). In control rats, Δ[Ca^2+^]_i_ peak was followed by a slow decay, whereas HIV-1 Tg rats display a long, persistent increase in neuronal Δ[Ca^2+^]_i_. **(B)** HIV-1 Tg rats had a greater reduction in total hemoglobin (Δ[HbT]) as well as **(C)** a rapid decrease in oxygenated hemoglobin (Δ[HbO_2_]) followed by a slight recovery from -30% to -20% that remained decreased over 60 min. **(D)** Memantine-pretreated HIV-1 Tg rats had a similar Δ[Ca^2+^]_i_ response to cocaine as control animals. However, control animals returned to baseline where HIV-1 Tg animals did not. **(E)** Time courses of control and HIV-1 Tg rat responses to cocaine showed similar temporal profiles in Δ[HbT] reductions. **(F)** Cocaine-induced Δ[HbO_2_] peak reduction was larger in HIV-1 Tg than in control rats. However, they returned to baseline at the same time. **(G–I)** HIV-1 Tg rats accumulated more intracellular neuronal Ca^2+^ and demonstrated significantly greater reductions in total and oxygenated hemoglobin. These enhanced responses were significantly reduced following memantine pretreatment in HIV-1 Tg. Green shaded area: no significant difference between curves. Red shaded area denotes significant differences between curves. Yellow area indicates the area quantified for the average rate of change for Δ[Ca^2+^]_i_, Δ[HbT], and Δ[HbO_2_]. *: *p* < 0.05, significant difference.

Figure 9 summarizes the comparison of cocaine’s effects on neuronal [Ca^2+^]_i_ and hemodynamic changes in PFC between control and HIV-1 Tg animals with vehicle and memantine pretreatment. As shown in Figure 9A, the time course of ∆[Ca^2+^]_i_ in the PFC of saline pretreated HIV-1 Tg animals showed a persistent increase over 60 min, whereas in controls, it reached a peak at ∼10 min after cocaine followed by a slow recovery. HIV-1 Tg rats pretreated with memantine displayed a similar neuronal Δ[Ca^2+^]_i_ response to cocaine as control rats (from t = −5 min to t = 45 min, p_-5 to 45_>0.05, green shaded area) (Figure 9D). However, the ∆[Ca^2+^]_i_ fluorescence in controls returned to baseline at t = 47.88 ± 10.53 min but not in HIV-1 Tg animals. A two-way ANOVA examining the interaction of drug (pretreatment with saline v memantine) and animal (control v HIV-1 Tg) for ∆[Ca^2+^]_i_ found no significant interaction between memantine treatment and animals (F(16,1) = 2.409, *p* = 0.145). The significant main effects were found for both memantine (*p* = 0.02) and animal (*p* = 0.042) on ∆[Ca^2+^]_i_. Post hoc analysis revealed that the mean neuronal ∆[Ca^2+^]_i_ was significantly higher in HIV-1 Tg rats pretreated with saline (∆[Ca^2+^]_i_ = 7.96 ± 1.6 (%/min), *n* = 5) than in controls (∆[Ca^2+^]_i_ = 3.46 ± 0.8 (%/min), *n* = 5, *p* = 0.01; Figure 9G). The mean rate of accumulated ∆[Ca^2+^]_i_ did not differ between controls (∆[Ca^2+^]_i_ = 2.18 ± 0.6 (%/min)) and HIV-1 Tg rats (3.01 ± 0.57 (%/min), *p* = 0.654). Pretreatment of HIV-1 Tg rats with memantine significantly reduced the accumulated Δ[Ca^2+^]_i_ following cocaine exposure (*p* = 0.008; Figure 9G).

In addition, the total cerebral volume in the PFC of HIV-1 Tg animals pretreated with saline decreased to −15% within t = 5 min after cocaine and remained reduced over the time of recording (60 min; Figure 9B); whereas in controls, Δ[HbT] decreased to −5% at t = 5 min and also remained reduced until the end of recording (Figure 9B). The time courses of hemodynamic response to cocaine for Δ[HbT] for memantine pretreated control and HIV-1 Tg rats showed no significant differences (Figure 9E). A two-way ANOVA of ∆[HbT] found only a simple main effect of drug pretreatment of the total cerebral blood volume (*p* = 0.006). The mean amplitude of Δ[HbT] was 2-fold larger in saline pretreated HIV-1 Tg PFC (−15.49 ± 3.2 (%/min)) than in controls (−7.0 ± 0.78 (%/min), *p* = 0.02). Cocaine-induced mean Δ[HbT] reductions in control and HIV-1 Tg animals were −1.87 ± 0.3 (%/min) and −4.04 ± 3.01 (%/min), respectively, and were not significantly different (*p* = 0.584; Figure 9H). Similar to ∆[Ca^2+^]_i_, a reduction in the total blood volume was attenuated in HIV-1 Tg rats pretreated with memantine when compared to saline pretreatment (*p* = 0.005).

HIV-1 Tg rats pretreated with saline had a rapid decrease in oxygenated hemoglobin followed by a slight recovery from −30% to −20% that remained over 60 min (Figure 9C). Cocaine-associated reduction in Δ[HbO_2_] that peaked at t = 4 min (Figure 9F) was larger in HIV-1 Tg rats (−23.48 ± 4.02 (%)) than in controls (−5.95 ± 0.64 (%), *p* = 0.015), but the returned time to baseline was similar (control: 38.33 ± 1.67 min and HIV-1Tg: 34.93 ± 12 min, *p* = 0.821; Figure 9F). A two-way ANOVA of ∆[HbO_2_] found no significant interaction between pretreatment and animal (F16,1) = 4.556, *p* = 0.052). Only the simple main effect of pretreatment was found to be significant (*p* = 0.002). The mean reduction in Δ[HbO_2_] was greater for HIV-1 Tg rats (−18.7 ± 1.6 (%/min)) than for controls (−6.12 ± 0.76 (%/min), *p* = 0.003; Figure 9I). The mean reduction in Δ[HbO_2_] following memantine pretreatment did not differ between the two groups (*p* = 0.863; Figure 9I). Memantine pretreatment of HIV-1Tg rats significantly attenuated Δ[HbO_2_] reduction (*p*<0.001).

## Conclusion and Discussion

HAND is likely due in part to the HIV-induced neurotoxicity associated with HIV reservoirs in the CNS, which can be exacerbated with cocaine exposure and vice versa. To our knowledge, this is the first study that looks at the effects of cocaine on the brain of a rodent model of neuroHIV that used cutting-edge imaging approach to simultaneously demonstrate the neuronal and hemodynamics responses to cocaine in the context of neuroHIV. Here, we investigated neuroHIV-associated synergistic potentiation of cocaine-induced neuronal and hemodynamic dysregulation in the PFC of rats, as well as the ability of NMDAR blockade (by memantine) to attenuate these deleterious effects. In wild-type (non-Tg) rats, memantine significantly shortened the duration of cocaine-induced neuronal calcium increase and attenuated the cocaine-associated reduction in brain tissue oxygenation and total blood volume. Memantine pretreatment of HIV-1 Tg rats also depressed the amplitude of the neuronal calcium increase and its long persistence while attenuating the reductions in tissue oxygenation and total blood volume from cocaine. When compared to non-Tg rats, HIV-1 Tg rats were significantly more reactive to cocaine-induced dysregulation of neuronal activity and hemodynamics. Pretreatment of both non-Tg and HIV-1 Tg rats with memantine significantly reduced the differences in cocaine response between them, suggesting that NMDAR blockade may blunt neuroHIV-associated PFC neuronal hyper-reactivity that is enhanced further by cocaine. Importantly, this result is similar to findings in our earlier studies, showing that the blockade of voltage-gated L-type calcium channels (L-channels) significantly reduces cocaine- and/or neuroHIV-induced PFC neuronal calcium and hemodynamic dysregulation (Nasif et al., 2005b; Ford et al., 2009; Khodr et al., 2016; Wayman et al., 2016; Du et al., 2021).

Consistent with our previous findings, when drug-naïve rats were exposed to cocaine, there was a rapid increase in calcium concentration in neurons in PFC (Allen et al., 2019; Du et al., 2021), especially in glutamatergic pyramidal neurons (Nasif et al., 2005a; Nasif et al., 2005b; Ford et al., 2009; Napier et al., 2014; Wayman et al., 2015; Wayman et al., 2016). Excessive calcium influx could be one of the triggers for excitotoxicity that contributes to dysregulation, injury, and ultimately loss of cortical neurons in mice and rats treated chronically with cocaine (Olney et al., 1991; George et al., 2008; Clare et al., 2021). Various mechanisms through which calcium influx induces excitotoxicity have been suggested, including NMDA receptor overactivation (Dodt et al., 1993; Colwell and Levine, 1996). In NDMA-mediated excitotoxicity, the sustained calcium influx stimulates intracellular cascades that ultimately lead to the activation of proteases and nucleases. Increased cytosolic positive charge (Ca^2+^) from the calcium influx is initially buffered by mitochondria. However, mitochondria eventually fail to regulate [Ca^2+^]_in_, leading to abnormal membrane depolarization and failure of ATP creation, decreased anti-oxidate production, and release of caspases furthering neuronal death (Armada-Moreira et al., 2020).

Rats in the context of neuroHIV displayed a significantly elevated PFC neuronal calcium influx, and this was further enhanced when exposed to cocaine as compared to control rats (∼2-fold). Previous studies into understanding the mechanism through which HIV exacerbates calcium influx point to HIV proteins including Tat and the envelope protein Gp120 as major contributors (Buch et al., 2011; Hu, 2016). Tat has been linked to upregulating tumor necrosis factor alpha, which enhances calcium influx in rat hippocampal pyramidal neurons and leads to the promotion of the cAMP/PKA pathway, which can increase the phosphorylation of NMDA receptors and calcium influx (Zidovetzki et al., 1998; Jara et al., 2007). Moreover, we also demonstrate the dysfunction of voltage-gated Ca^2+^ channels (VGCCs) induced by Tat. For instance, Tat not only significantly enhances calcium influx through VGCCs (Napier et al., 2014) but also increases the expression of L-channel VGCCs in mPFC pyramidal neurons (Wayman et al., 2012). In addition, in conjunction with Tat, Gp120 has been shown to work synergistically with Tat, potentiating its calcium influx even when levels of Tat were subtoxic (Nath et al., 2000). Astrocytes, which clear 90% of extracellular glutamate, are also impacted by Tat, which inhibits their ability to remove extracellular glutamate, the main ligand of NMDA receptors (Kojima et al., 1999; Zhou et al., 2004). Collectively, all of these changes to normal cellular physiology likely contribute to Ca^2+^-induced neuronal excitotoxicity and ultimately to the mechanism of HAND.

Pretreatment with memantine not only significantly attenuated calcium influx in this rat model of neuroHIV but also attenuated the influx in non-Tg control rats, and this was associated with a return of intracellular calcium back to baseline levels within 60 min. When we compared the calcium transients of the memantine-pretreated control rats to the memantine-pretreated HIV-1 Tg rats, we found that there was no significant difference between the curves. This suggests that the blockade of overactive NMDARs was able to diminish the HIV-induced abnormal increase of neuronal calcium influx. Taken together, our novel findings support that, in combination with the selective blockade of overactive L-type calcium channels, antagonizing hyperactive NMDARs by memantine has a potential to be used clinically to decrease neuronal excitotoxicity in both HIV(+) and HIV (-) cocaine users, with the possibility of substantially more benefit to those individuals who are HIV(+) (Khodr et al., 2016). Given that previous clinical trial studies failed to significantly decrease neurocognitive deficits in patients with HAND, likely due to *specific* blockade of NMDARs (Schifitto et al., 2007) or L-channels (Navia et al., 1998), the combined treatment with selective NMDA antagonist and L-type calcium channel blocker may over time lead to reducing cocaine-induced excitotoxicity and limiting neuronal degeneration in the brain regions that regulate neurocognition. Further work is required to validate this novel concept and hypothesis (Hu, 2016; Du et al., 2021).

The ability of memantine to treat drug addiction remains understudied. Preclinically, memantine produces different results depending on the species. For example, pretreatment with the NMDA antagonist in rats led to a reduction in drug intake in a self-administering mouse model, whereas it increased drug intake in self-administering monkeys (Blokhina et al., 2005; Newman and Beardsley, 2006). On the other hand, clinically, memantine alone has shown no benefit in the treatment of cocaine use disorders as it does not reduce craving or cocaine consumption (Collins et al., 2006; Bisaga et al., 2010). However, these studies were limited in duration (range: 47 days to 12 weeks) and failed to assess additional complications of chronic cocaine use such as its impact on neurotoxicity. Furthermore, there was no combined L-channel blockade in these studies, and this could be a potential but very crucial reason.

Besides cocaine- and neuroHIV-induced functional alterations in the PFC *per se*, PFC neuronal and hemodynamic dysregulation demonstrated in the present study might also be affected by the dysfunction of the mesocorticolimbic dopamine (DA) system. Previous studies have reported that similar to cocaine, Tat also significantly inhibits the function of DA transporters (DATs), which abnormally decreases DA reuptake, promotes DA neurotransmission, and prolongs/enhances the effects of DA on postsynaptic brain regions (Zhu et al., 2018; Quizon et al., 2021). Additionally, in HIV-1 Tg rats that express Tat and several other HIV proteins, we previously revealed dysfunction in glutamatergic PFC pyramidal neurons (Nasif et al., 2005a; Nasif et al., 2005b; Ford et al., 2009; Wayman et al., 2012; Napier et al., 2014; Wayman et al., 2015; Wayman et al., 2016; Allen et al., 2019; Du et al., 2021) and GABAergic (dorsal/ventral) medium spiny striatal neurons (Chen et al., 2018), which are both influenced by DA as they are targets of DA terminals. The consequential effects of such DAT dysfunction may abnormally increase the functional activity/expression of L-channels and/or NMDARs, thereby jointly causing neuronal and hemodynamic dysregulation in the brain regions that regulate neurocognition. In combination with such alterations in these neurons *per se*, these neuronal dysfunctions associated with and in response to changes in DA neurotransmission may also contribute to the mechanism of HAND, as well as that underling the comorbidity of HAND and cocaine use disorders.

Both the naïve wild-type and HIV transgenic rats displayed significant hypoxemia (Δ[HbO_2_]) and reduction in cerebral blood volume (Δ[HbT]) following acute cocaine. While the decreases in both of these parameters is consistent with our previous reports as well as with clinical findings, the HIV 1-Tg rat reduction was more severe (2-fold) (Volkow et al., 1988; Chen et al., 2016; Allen et al., 2019; Du et al., 2020). Pretreatments with memantine in HIV 1-Tg rats abolished this substantial hypoxia, as demonstrated by the time-course imaging and quantification of oxygenated hemoglobin and total blood volume, which showed no significant differences with controls. This may be due to maintained neurovascular coupling as both blood volume and oxygenation return to baseline at the same time as neuronal activity.

Attenuation of blood flow in the PFC, a critical brain region that regulates neurocognition, has been observed in both chronic cocaine usage and HAND (Maini et al., 1990; Schielke et al., 1990; Allen et al., 2019). Referred to as hypofrontality, this reduction in blood flow might underlie decreases in higher-order cognitive functions such as learning and advanced planning (Volkow and Morales, 2015). Studies involving the use of memantine to treat diseases with reduced blood volume, such as stroke and Alzheimer’s disease, have demonstrated success in modifying hemodynamics and disease progression. In patients with Alzheimer’s disease, the addition of 20 mg/kg of memantine to donepezil (acetylcholinesterase inhibitor) significantly improved the mini mental status examination and clinical global impression improvement scale after 24 weeks. This increase in function relative to control was associated with more flow in the frontal gyri (Araki et al., 2014). Moreover, in animal models of stroke, memantine reduced the infarcted area and penumbra development (López-Valdés et al., 2021). These studies suggest that memantine may have a role in treating drug addiction and HAND by modifying the pathogenesis of the cerebral vasculature. Consistent with this hypothesis, an open-label study of 40 mg/kg of memantine that treated patients for 32 weeks found an improvement in their neurocognitive function (Zhao et al., 2010). Whether this hypothesized decrease in ischemia is due to NMDA antagonist’s effect on the brain’s vasculature directly or is a consequence of decreased overactivation of the neurons leading to decreased flow demands remains unclear and requires further investigation. However, attenuating ischemia and hypofrontality through NDMA antagonist such as memantine may slow the onset and progression of cognitive decline.

Although the present study provides insight into the pathophysiology of HIV and cocaine-induced neurotoxicity, there are some limitations. This study is limited by its small sample size and use of only male rats. Despite the limited sample, cocaine induced large, repeatable results in both the wild-type and HIV-1 Tg rats, which allowed us to document significant effects of HIV and blockade of overactive NMDARs on cocaine-induced excessive neuronal calcium influx and hemodynamics. We selectively choose to study these dysfunctional alterations in male rats as sex hormones can affect the cerebral vasculature (Duckles and Krause, 2007). However, studies continue to demonstrate that sexes respond to cocaine differently (Clare et al., 2021). Therefore, future studies should investigate the impact of sex on cocaine-induced changes in HIV. Lastly, to obtain these results, all animals were anesthetized during experimentation. It is possible that our outcomes could have been influenced by interactions between the anesthetic and the compounds used. Also, our animals were individually housed, and further studies are needed to determine the generalizability of our findings to animals reared in group housing.

In summary, in the present study, we have demonstrated the use of a custom imaging modality to acquire simultaneous information regarding the pathophysiology of neuronal and hemodynamic responses in the brain of a rat model of neuroHIV exposed to cocaine. Moreover, our novel findings also indicated that the blockade of overactive NMDARs with memantine attenuated the exaggerated calcium influx and hypoxia seen in the context of neuroHIV, either with or without concurrent exposure to cocaine. Thus, this research provides new evidence to support our perspective that, in combination with the appropriate blockade of overactivation/overexpression of L-type calcium channels, antagonism of overactive NMDARs in hyperactive PFC pyramidal neurons may be a useful and effective novel therapeutic approach for treating neuroAIDS-associated or cocaine-induced neurotoxicity, as well as the comorbidity of HAND and cocaine use disorders.

## Data Availability

The raw data supporting the conclusion of this article will be made available by the authors without undue reservation.
