# Supplementary material for: Memantine Attenuates Cocaine and neuroHIV Neurotoxicity in the Medial Prefrontal Cortex
Source: Front Pharmacol. 2022 May 25;13:895006. doi: 10.3389/fphar.2022.895006 (PMC9174902; doi:10.3389/fphar.2022.895006)
Supplement: Supplementary file 1 [file DataSheet1.docx]

Supplemental Materials:


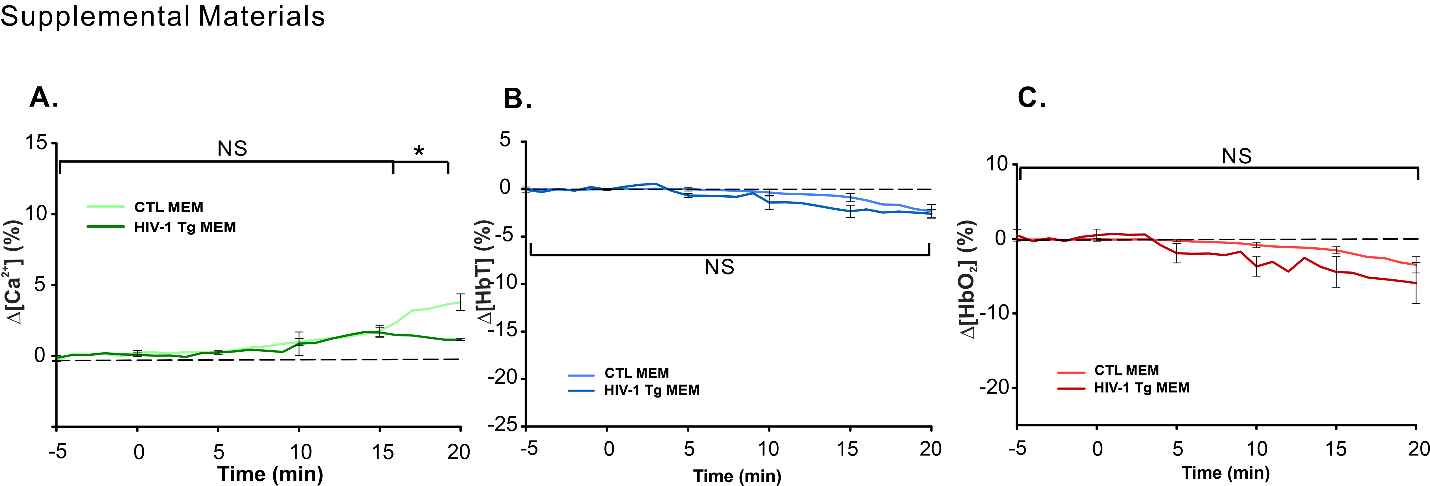


**Figure S1:** Time course of **A)** Ca^2+^, **B)** total hemoglobin (HbT), and **C)** oxygenated hemoglobin (HbO_2_) in control F344 rats and HIV-1 Tg rats exposed to NMDA antagonist memantine.


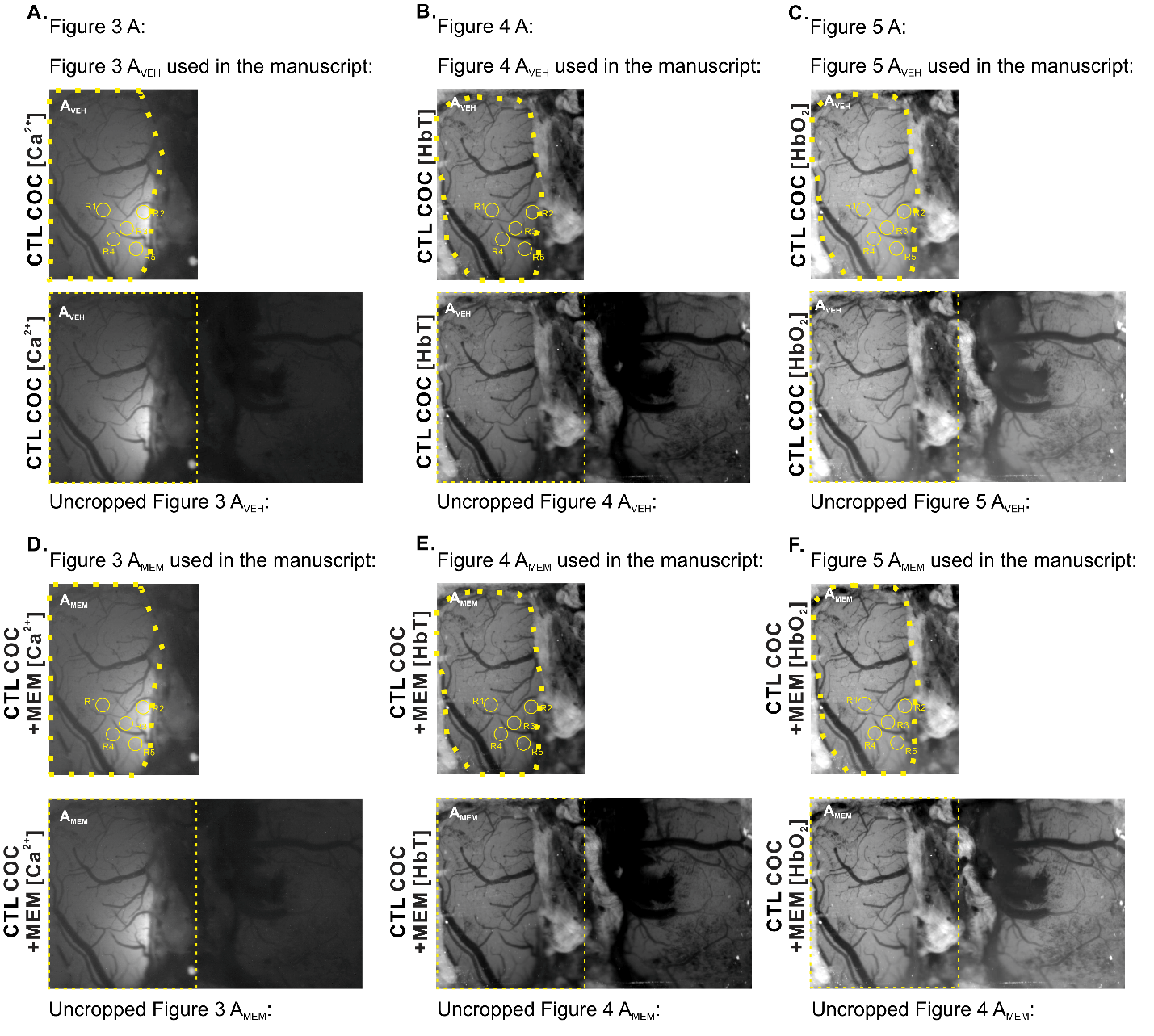
**Figure S2:** **A.)** Ca^2+^ fluorescence **B.)** total hemoglobin (HbT), and **C)** oxygenated hemoglobin (HbO_2_) representative images demonstrating the changes in left half PFC where has GCaMP6f injection, and its uncropped full PFC image for the control rat under cocaine (COC) stimulation without or with **D-F)** memantine (MEM).


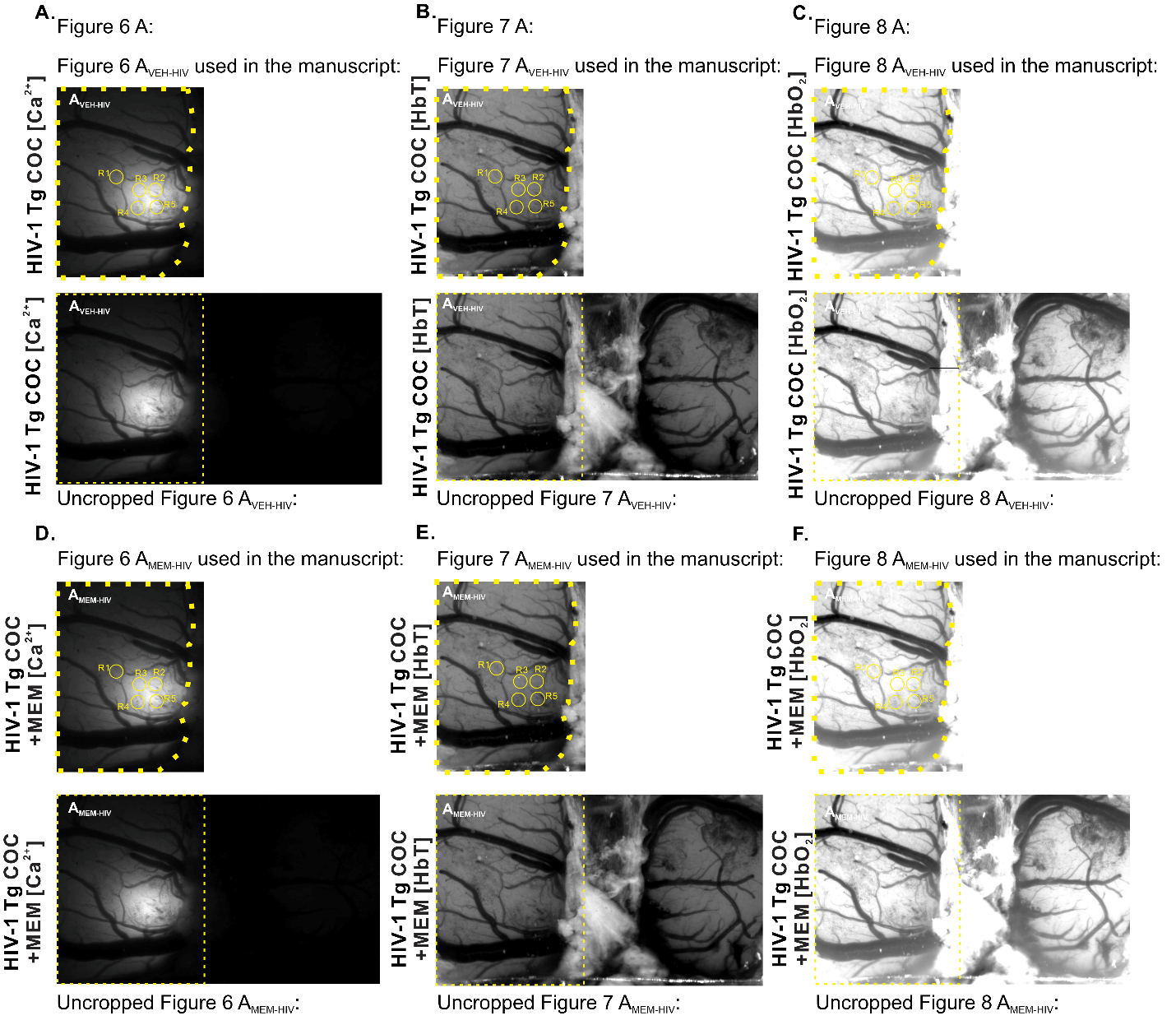


**Figure S3:** **A.)** Ca^2+^ fluorescence **B.)** total hemoglobin (HbT), and **C)** oxygenated hemoglobin (HbO_2_) representative images demonstrating the changes in left half PFC where has GCaMP6f injection, and its uncropped full PFC image for the HIV-1 Tg rat under cocaine (COC) stimulation without or with **D-F)** memantine (MEM).

**Table S1: p Values Comparison Between Control and HIV-1 Tg rats (Figure 2), Including Cumulative** $\Delta$ **[Ca^2+^],** $\Delta$ **[HbT], and** $\Delta$ **[HbO_2_] Memantine Responses over the baseline**

|  | CTL: Baseline vs. After MEM | CTL: Baseline vs. After MEM | CTL MEM vs. HIV-1 Tg MEM |
| --- | --- | --- | --- |
| $\Delta$[Ca^2+^]  p-value | p=0.006 | p=0.005 | p=0.081 |
| $\Delta$ [HbT]  p-value | p= 0.151 | p= 0.061 | p= 0.091 |
| $\Delta$[HbO_2_]  p-value | P = 0.051 | p= 0.105 | p= 0.39 |

**Table S2: One-way ANOVA Comparison between Control and HIV-1 Tg rats of integrated Changes Response (%/min) to Cocaine with or without Memantine, Including** $\Delta$ **[Ca^2+^],** $\Delta$ **[HbT], and** $\Delta$ **[HbO_2_].**

|  | Group | Integration (%/min) | p-value |
| --- | --- | --- | --- |
| $\Delta$[Ca^2+^]  p-value | CTL COC | 3.464±1.758 | p= 0.303 |
|  | CTL COC MEM | 2.181±1.057 |  |
|  | HIV-1 Tg COC | 7.958±3.710 | p= 0.039 |
|  | HIVT-1 Tg COC+MEM | 3.014±1.139 |  |
| $\Delta$[HbT]  p-value | CTL COC | -7.009±1.753 | p= 0.003 |
|  | CTL COC MEM | -1.874±0.530 |  |
|  | HIV-1 Tg COC | -15.488±7.228 | p= 0.039 |
|  | HIVT-1 Tg COC+MEM | -4.037±6.024 |  |
| $\Delta$[HbO_2_]  p-value | CTL COC | -6.125±1.688 | p=0.004 |
|  | CTL COC MEM | -1.230±0.843 |  |
|  | HIV-1 Tg COC | -18.702±3.612 | p=0.013 |
|  | HIVT-1 Tg COC+MEM | -1.981±10.659 |  |

**Table S3: One-way ANOVA Comparison between Control and HIV-1 Tg rats of Peak Changes Response (%) to Cocaine with or without Memantine, Including** $\Delta$ **[Ca^2+^],** $\Delta$ **[HbT], and** $\Delta$ **[HbO_2_].**

|  | Group | Integration (%/min) | p-value |
| --- | --- | --- | --- |
| $\Delta$[Ca^2+^]  p-value | CTL COC | 4.465±2.063 | p= 0.967 |
|  | CTL COC MEM | 4.406±1.352 |  |
|  | HIV-1 Tg COC | 6.713±1.930 | p= 0.019 |
|  | HIVT-1 Tg COC+MEM | 3.317±1.268 |  |
| $\Delta$[HbT]  p-value | CTL COC | -6.214±1.508 | p= 0.096 |
|  | CTL COC MEM | -4.213±1.118 |  |
|  | HIV-1 Tg COC | -14.828±5.399 | p= 0.077 |
|  | HIVT-1 Tg COC+MEM | -6.947±5.994 |  |
| $\Delta$[HbO_2_]  p-value | CTL COC | -7.623±1.869 | p=0.213 |
|  | CTL COC MEM | -5.951±1.104 |  |
|  | HIV-1 Tg COC | -33.968±5.152 | p=0.048 |
|  | HIVT-1 Tg COC+MEM | -23.476±8.032 |  |

**Table S4: Two-way-Anova of Comparison between Control and HIV-1 Tg rats of Cumulative Changes Response to Cocaine with or without Memantine, Including** $\Delta$ **[Ca^2+^],** $\Delta$ **[HbT], and** $\Delta$ **[HbO_2_].**

|  | CTL COC vs. CTL COC+MEM | HIV-Tg COC vs. HIV-1 Tg COC+MEM | CTL COC vs. HIV-1 Tg COC | CTL COC+MEM vs. HIV-1 Tg COC+MEM |
| --- | --- | --- | --- | --- |
| $\Delta$[Ca^2+^]  p-value | p= 0.473 | p= 0.008 | p= 0.01 | p=0.654 |
| $\Delta$[HbT]  p-value | p= 0.187 | p= 0.005 | p= 0.02 | p= 0.584 |
| $\Delta$[HbO_2_]  p-value | p= 0.252 | p<0.001 | p= 0.003 | p= 0.863 |
